# Genomic Surveillance Uncovers a Shift in Respiratory Syncytial Virus Subtype Dominance and the Local Expansion of Subclade B.D.E.1.7

**DOI:** 10.1093/ofid/ofag564

**Published:** 2026-09-09

**Authors:** Emily LaVerriere, Sasha Behar, Yan Mei Liang, Marisa Nielsen, Manish Sagar, John H Connor

**Affiliations:** Department of Virology, Immunology and Microbiology, Boston University Chobanian & Avedisian School of Medicine, Boston, Massachusetts, USA; National Emerging Infectious Diseases Laboratories, Boston University, Boston, Massachusetts, USA; Department of Virology, Immunology and Microbiology, Boston University Chobanian & Avedisian School of Medicine, Boston, Massachusetts, USA; National Emerging Infectious Diseases Laboratories, Boston University, Boston, Massachusetts, USA; Department of Virology, Immunology and Microbiology, Boston University Chobanian & Avedisian School of Medicine, Boston, Massachusetts, USA; Department of Medicine, Boston University Chobanian & Avedisian School of Medicine, Boston, Massachusetts, USA; Department of Medicine, Boston University Chobanian & Avedisian School of Medicine, Boston, Massachusetts, USA; Clinical Microbiology, Boston Medical Center, Department of Pathology and Laboratory Medicine, Boston University Chobanian & Avedisian School of Medicine, Boston, Massachusetts, USA; Department of Virology, Immunology and Microbiology, Boston University Chobanian & Avedisian School of Medicine, Boston, Massachusetts, USA; Department of Medicine, Boston University Chobanian & Avedisian School of Medicine, Boston, Massachusetts, USA; Department of Virology, Immunology and Microbiology, Boston University Chobanian & Avedisian School of Medicine, Boston, Massachusetts, USA; National Emerging Infectious Diseases Laboratories, Boston University, Boston, Massachusetts, USA

**Keywords:** Boston, genomics, Massachusetts, molecular epidemiology, phylogenetic analysis, respiratory syncytial virus, respiratory syncytial virus infections, respiratory syncytial viruses, viruses

## Abstract

**Background:**

Respiratory syncytial virus (RSV) is a leading cause of hospitalization among children under 5 and adults over 60 due to its ability to cause acute respiratory illness. Recent approvals of vaccines and monoclonal antibodies against RSV have raised concerns about the potential of prophylactic-evading viral mutations.

**Method:**

We analyzed 126 RSV-positive nasopharyngeal swabs from Boston Medical Center during the 2024–2025 respiratory season via amplicon-based whole-genome sequencing. We identified nucleotide variants, assigned clades, and built maximum-likelihood phylogenetic trees to examine genetic changes and evolution.

**Results:**

Out of the 126 genomes analyzed, 94 (74.6%) were RSV-A subtype, indicating a return to RSV-A dominance in the region. Within the RSV-B genomes, a large minority belong to the recently defined B.D.E.1.7 subclade (n = 12, 37.5% of RSV-B samples), which has only appeared sporadically in other US states. Mutations associated with monoclonal antibody or vaccine resistance were not observed.

**Conclusions:**

These findings show that in the Boston metropolitan area, RSV subtype dominance continues to flip between seasons. Investigation of the clades within both subtypes did not reveal evidence of any virus seeding from the preceding season. Though no mutations associated with prophylactic resistance were identified, surveillance of RSV remains critical to study evolution and transmission dynamics, particularly at the local level.

## Teaser

Surveillance of seasonal respiratory syncytial virus (RSV) remains critical to tracking evolution and identifying anti-prophylactic resistance. A total of 126 RSV genomes from Boston Medical Center generated from 2024–2025 season samples reveal a shift to RSV-A dominance and the regional expansion of the B.D.E.1.7 subclade.

Respiratory syncytial virus (RSV) remains a prominent cause of acute respiratory disease in the United States and worldwide [1, 2]. At the extremes of the age spectrum, infection with RSV poses an elevated risk of developing into severe disease: annually in the United States, RSV is responsible for approximately 80 000 hospitalizations in infants under the age of 5 [3] and 159 000 hospitalizations in adults over 65 [4, 5]. Infection in infants has been linked with the development of asthma and wheezing into adulthood [6]. In older adults, infection may exacerbate preexisting asthma or chronic obstructive pulmonary disease [7]. Though mortality is low in infants [1], RSV in-hospital mortality is estimated to be as high as 17% in adults over 65 [8].

Current interventions for RSV are centered on prophylaxis with vaccines or monoclonal antibodies that have been shown to reduce RSV morbidity and mortality. These countermeasures target the RSV fusion (F) protein due to its high conservation across RSV strains [9]. Although there are currently no known vaccine escape mutations identified in RSV, we have seen the development of such mutations in other viruses in recent history, and therapeutic antibody-evasion mutations in RSV have been recently detected on a variety of genetic backgrounds [10].

RSV evolution can also impact the ongoing development of monoclonal antibodies; this has been illustrated by a Phase III clinical trial for suptavumab [11]. In this trial, ongoing evolution altered the epitope targeted by the therapeutic candidate antibody, creating natural resistance mutations in the trial population, which led to loss of therapeutic efficacy in some RSV infections [11]. Though future evolution of RSV is difficult to predict, this experience highlights the importance of maintaining ongoing genomic surveillance to identify the appearance of prophylactic-evading mutations.

RSV comprises 2 subtypes, RSV-A and RSV-B. These subtypes share approximately 90% nucleotide sequence identity [12] but are antigenically distinct due to larger differences in their glycoprotein sequence [13]. These differences result in differential host interactions between subtypes, resulting in similar but distinct evolutionary paths. In the past 2 decades, both subtypes have acquired unique partial duplications within their glycoprotein gene that have led to the extinction of prior circulating variants [14, 15]. Clinically, recent investigations conclude that morbidity and mortality are similar between subtypes [16].

Routine RSV genomic surveillance has increased across the United States since 2020. Studies covering locales such as Minnesota [17], Maryland [18], Michigan [19], and Connecticut [20] have laid the groundwork to track viral evolution throughout the country over the last several respiratory seasons. Globally, similar efforts have expanded in a variety of countries [21–24], as well as through the WHO surveillance program [25], making analysis of global transmission patterns more feasible. Notably, these studies have suggested that transmission is strongly enabled by rapid global transport (eg, plane travel), enabling RSV transmission.

In addition to genomic surveillance's impact on understanding transmission on a global scale, it has had an equally important influence on understanding RSV transmission dynamics within small geographic regions. This has included the recognition of switching subtype dominance from RSV-A in 2022 to RSV-B in 2024 in the Northeast United States [26, 27] and the detection of clades that are minor worldwide but play an outsized role in local transmission. Sequencing studies in Ireland led to the definition of the A.D.1.4 clade [28], and recent surveillance in Maryland [18] revealed the dominance of the A.D.1.6 clade over a period of time in which other US sites detected very few A.D.1.6 genomes.

Here, we report recent genomic surveillance of 126 RSV-positive diagnostic samples from a large safety-net hospital in the Northeastern United States. This sequencing uncovered a significant change in subtype dominance from B to A over 2 seasons. Additionally, we highlight the expansion of a single subclade of RSV-B, notable within this sample set and a complementary local sample set, but not at other RSV sequencing sites around the United States. This subclade has expanded significantly worldwide since 2024, and the increased genomic surveillance allows the tracking of its expansion across multiple continents.

## METHODS

### Collection of Samples and Patient Data

Nasopharyngeal swabs were sourced from Boston Medical Center (BMC) patients and screened for respiratory viruses using the bioMérieux BIOFIRE® Respiratory 2.1 Panel; Cepheid Xpert® Xpress CoV-2/Flu/RSV plus panel; Diasorin Simplexa® COVID-19 & Flu A/B Direct; bioMérieux BIOFIRE® SPOTFIRE® Respiratory/Sore Throat (R/ST) Panel; or the Roche Diagnostics cobas® Liat SARS-CoV-2, Influenza A/B & RSV panel, then stored in viral transport medium at 4°C. All swabs positive for RSV between July 2024 and May 2025 were requested from the BMC Clinical Microbiology Laboratory, and approximately one-third of the total RSV-positive swabs were received and transferred to the National Emerging Infectious Diseases Laboratories, where they were frozen at −80°C until processing. The remaining samples could not be received due to being already discarded, of inadequate quantity, or missing. Discard swabs positive for RSV from January through June 2024 were previously gathered following the same methods, detailed in our previous study [27]. Ct values were not available for the samples provided.

### Preparation of Samples for Next-Generation Sequencing

Briefly, isolated genomes were RNA extracted and reverse-transcribed, then amplified using RSV subtype-specific amplicon primer sets [29]. Successful amplifications were determined through agarose gel validation and then used to generate DNA libraries with the NEBNext UltraExpress FS DNA Library Prep Kit from New England Biolabs. Resulting samples were sequenced as 150-bp paired-end reads on an Illumina NextSeq 2000 P2 chip for 800 million total reads, with a 20% PhiX spike-in. Information regarding patient age and sex was identified from the electronic medical record. All sample and data collection was approved by the Boston University Institutional Review Board (approval number: H-42887, minimal-risk approval with consent waiver).

### Sequencing Data Processing

We aligned samples to the reference genomes for RSV-A (hRSV/A/England/397/2017, Global Initiative on Sharing All Influenza Data [GISAID] [30]: EPI_ISL_412866) and RSV-B (hRSV/B/Australia/VIC-RCH056/2019, GISAID: EPI_ISL_1653999) using bwa [31]. We trimmed primer-binding sites, assessed coverage, generated consensus sequences, and called variants with samtools and iVar [32, 33]. Consensus sequences required a minimum read depth of 20, a minimum quality score of 20, and a call fraction above 50%. Variants called required a minimum quality of 20. Median depth across the genome and percentage of nucleotides with at least 20× depth per genome are reported in Supplementary Table 1.

We processed all consensus genomes through Nextclade [34] to assign clades [35] and assess additional quality metrics. For all downstream analyses, we required consensus genomes to have an overall quality in Nextclade other than “bad,” a minimum of 20× coverage over at least 90% of the full genome and over each of the F and G genes. We manually examined 24 genomes with a Nextclade quality of “mediocre,” excluding 21 based on misalignments or private mutations. Clades were assigned using lineage definitions from September 2025.

### Comparison With Other Datasets

We identified other US sites with RSV surveillance over the 2024–2025 season. For Minnesota and Maryland, we included the proportions of RSV-A to RSV-B genomes sequenced as reported in publications [17, 18]. We also searched within RSV genomes available through Pathoplexus in November 2025. We filtered to genomes collected between 1 October 2024 and 30 September 2025, from the country “USA”, with a minimum length of 14 000 bp. We then included all submitting groups with at least 30 RSV genomes meeting these conditions. We updated this search in July 2026 for supplemental analysis.

We also searched for all B.D.E.1.7 genomes globally. In July 2026, we filtered Pathoplexus RSV-B genomes to those with a collection date from 1 October 2024 through 30 September 2025 and with the lineage B.D.E.1.7. We performed a similar search within GISAID, filtering to genomes with subtype B, collection date from 1 October 2024 through 30 September 2025, lineage B.D.E.1 with substitutions G:K32R and G:K223E (the lineage-defining mutations for B.D.E.1.7). We checked clade assignments with Nextclade and removed any non-B.D.E.1.7 genomes. We removed any duplicate genomes with the Pathoplexus dataset and assessed quality metrics of all comparator genomes with Nextclade, as described above. Genomes used are listed in the background set of Pathoplexus SeqSet PP_SS_3070.1 [36] and GISAID EPI_SET_260325ht (Supplementary Table 2).

### Phylogenetic and Selection Analyses

We performed multiple sequence alignment using MAFFT v7 [37]. We generated maximum-likelihood phylogenetic trees using IQTree v2.3.6 [38] with ModelFinder and ultrafast bootstrap approximation with nearest neighbor interchange [39]. We also used IQTree for time-resolved trees, using the least square dating method [40].

We performed selection analyses using HyPhy v2.5.86 [41] on an alignment of the G protein with any codons containing ambiguous nucleotide calls masked per genome. We ran both FEL (Fixed Effects Likelihood) [42] and MEME (Mixed Effects Model of Evolution) [43] on all BMC RSV-B genomes, with B.D.E.1.7 genomes set as foreground. All other settings were default, including a significance threshold of 0.1.

We plotted all data in R 4.5.1, with the tidyverse, Biostrings, RColorBrewer, and ggtree suite of packages [44–47]. We arranged final figures in Adobe Illustrator.

## RESULTS

### Sample Demographics are Consistent With Broader Regional Trends

For this study, we collected 244 discarded clinical samples that tested positive for RSV at the BMC Clinical Microbiology laboratory between July 2024 and May 2025 (Table 1). The majority of these samples were from patients aged 0–2 years; the median age was 2 years, with a range of 0–94 years. Samples were slightly more common from male patients (n = 133, 54.5%) than female patients (n = 111, 45.5%). These demographics are similar to those observed in the RSV-NET data nationally (Supplementary Figure 1). We attempted whole-genome sequencing on all of these samples, and 126 of these showed high-quality mapping after sequencing, data processing, and assessment of data quality (Supplementary Figure 2).

**Table 1.** Demographics of Samples Received Which Generated High-Quality Genomes

Variable	Level	Received	Generated High-Quality Genomes
Age, y	Range	0–94	0–94
	Median	2	2
	Mean ± SD	10 ± 20	10 ± 20
	0–2	137	76
	60–74	14	9
	≥75	5	3
Sex	Female	111	50
	Male	133	76
Collection month, 2024	July	1	0
	August	3	2
	September	1	1
	October	9	6
	November	67	37
	December	122	59
Collection month, 2025	January	22	14
	February	1	1
	March	15	4
	April	3	2
	May	0	0
Total for season	…	244	126

Demographics of swabs from July 2024 to May 2025 that generated high-quality genomes used for subsequent analysis.

**Figure 1.**
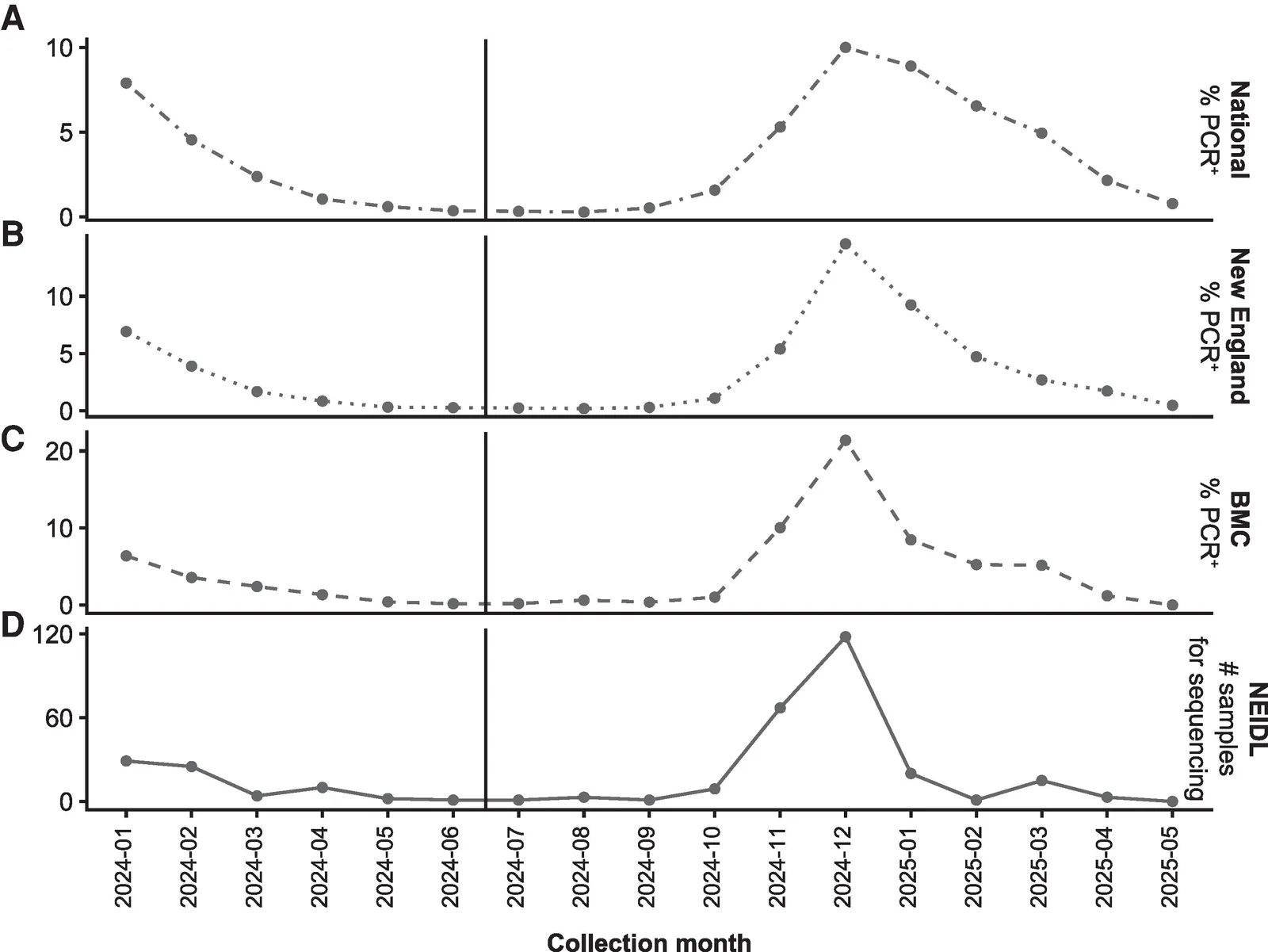
Proxies for RSV transmission from January 2024 through May 2025. (*A*, *B*) The “National” and “New England” panels show the average percent of RSV-PCR test positivity per month, according to RSV-NET data. (*C*) The “BMC” panel shows the percent of tests performed in the Clinical Microbiology lab per month that were positive for RSV. (*D*) The “NEIDL” panel shows the number of samples that were sent from BMC Clinical Microbiology to the NEIDL for sequencing, grouped by month of sample collection. The vertical line between June and July 2024 represents the transition point between the previously published 2024 samples and the newly sequenced 2024–2025 samples.

We next analyzed how our sample collection compared to disease detection locally and nationally. For this analysis, we included both the dataset described above and a second set of RSV+ samples that were collected at BMC from January 2024 to June 2024 [27]. The time period covered by the resulting dataset was January 2024 until May 2025. BMC tested 15 427 samples in this time window, of which 851 were positive for RSV (5.52%). There were 2 periods of high incidence of RSV nationally and in New England, as calculated from RSV-NET [48] data (Figure 1*A* and 1*B*). The percent of RSV+ samples per month for BMC closely correlated with New England and national percentages (Figure 1*C*), as did the number of samples collected for sequencing (Figure 1*D*), but we noted a more rapid fall off of polymerase chain reaction (PCR) positivity for both New England and BMC in January of 2025 compared to national trends.

### Regional Subtype Prevalence Returns to RSV-A

**Figure 2.**
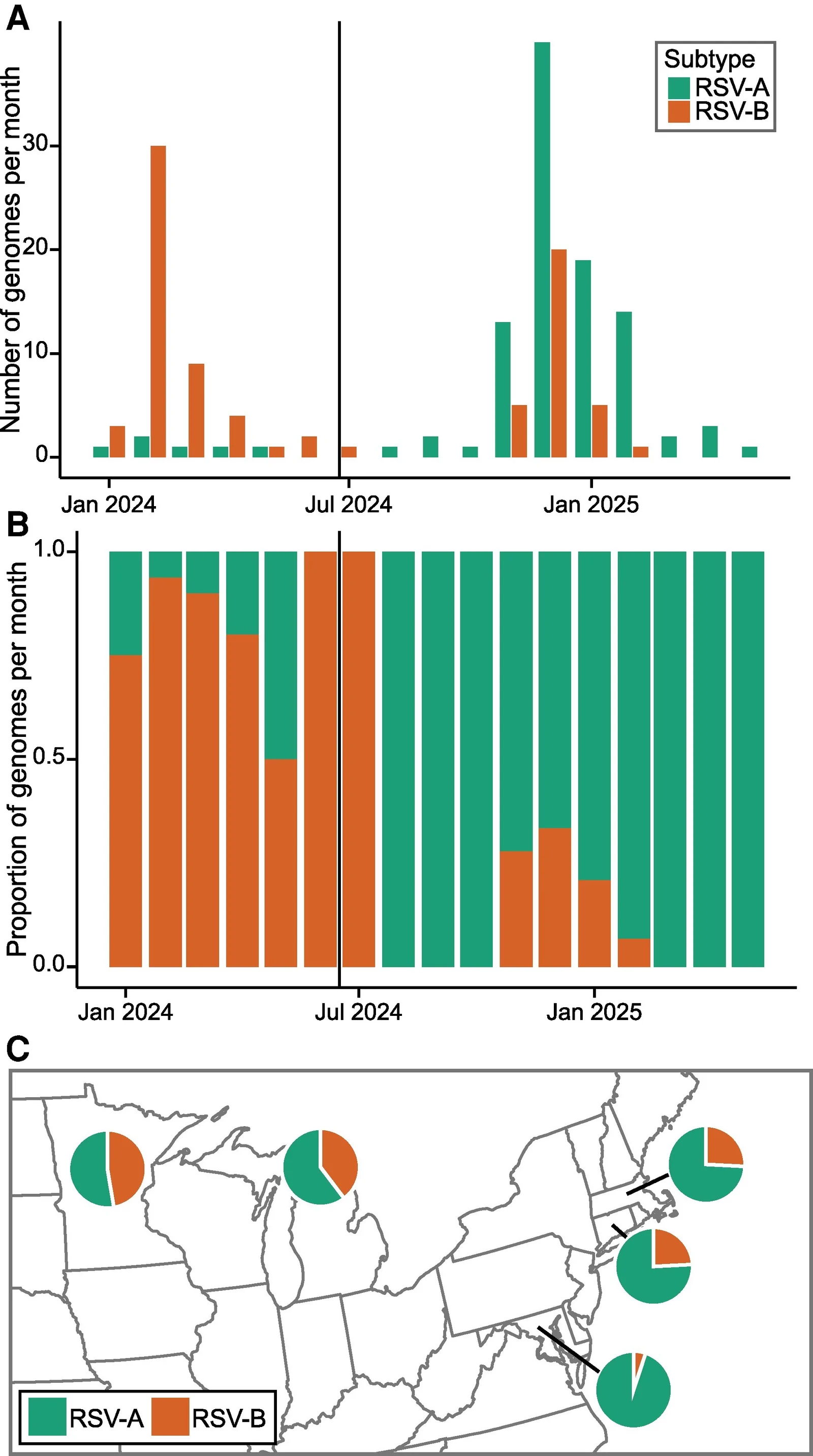
Temporal and spatial trends of RSV-A to RSV-B proportions. Comparison of the number of genomes sequenced and assigned to a subtype of RSV, grouped by sample collection month. (*A*) Total number of samples that generated high-quality genomes per month. (*B*) Proportion of subtype of samples that generated high-quality genomes per month. The vertical line between June and July 2024 represents the transition point between the previously sequenced 2024 samples [27] and 2024–2025 samples presented in this study. (*C*) Proportion of RSV-A and RSV-B genomes sequenced from samples collected in the 2024–2025 season across United States locations. Locations include Massachusetts (a combination of the BMC samples sequenced in this study and the Mass General Brigham [MGB] samples sequenced at the Broad Institute), Connecticut (Yale), Maryland (Johns Hopkins), Michigan (University of Michigan), and Minnesota (Minnesota Department of Public Health). Additional locations are included in Supplementary Table 3.

The prevalence of RSV-A and B genotypes identified from sequenced RSV genomes between January 2024 and May 2025 is shown in Figure 2*A*. As was noted previously, the January 2024–June 2024 analysis showed a predominance of RSV-B in circulation. In contrast, we identified a total of 94 RSV-A and 32 RSV-B new genomes between July 2024 and May 2025. This 3:1 ratio of RSV-A to B emphasizes the dramatic increase in total numbers of RSV-A genomes from January to June 2024. As highlighted in Figure 2*A*, RSV-B had dominated the samples sequenced from the end of the 2023–2024 season, while RSV-A genomes dominated the 2024–2025 season, which was especially notable at the beginning and end of the season (Figure 2*B*). This trend of RSV-A dominance in the 2024–2025 season was not limited to our analysis; it has also been observed in other east coast regions of the United States (Maryland [18], Connecticut [20]), while midwestern regions (Michigan, Minnesota [17, 19]) observed more equal proportions of RSV-A and RSV-B (Figure 2*C*). Of 13 states with at least 30 RSV genomes sequenced from the 2024–2025 season, 9 states showed RSV-A dominance (>60% RSV-A genomes), and 3 states had similar proportions of RSV-A and RSV-B (40%–60% RSV-A genomes). One state, Missouri, showed RSV-B dominance (>60% RSV-B genomes) (Supplementary Table 3).

### Monitoring Substitutions Within the F Protein

As the current vaccines and monoclonal antibodies involve key aspects of the F protein, we examined non-synonymous mutations present within known antigenic regions of the F gene (Table 2). One A.D.3.1 genome contained the K65R substitution, which has been previously identified to confer resistance to nirsevimab in combination with the F:I64M substitution in an RSV-B virus [49]. This substitution was also recently detected in Ireland surveillance [28]. We also identified 2 amino acid substitutions within the clesrovimab-binding site in F: I432V and K445R. Neither mutation has been reported to be associated with clesrovimab resistance, though other substitutions at site 445 affected clesrovimab neutralization in a recent deep mutational scanning study [50].

**Table 2.** Substitutions Observed Within Antigenic Regions of the F Gene

Site	Substitution	Number of Genomes			Lineage	Number of BMC Genomes Per Lineage With This Substitution	Percent of All BMC Genomes of Lineage With This Substitution
		Both Seasons	2023–2024	2024–2025			
65	K65R	1	0	1	A.D.3.1	1	25%
209	R209Q	3	3	0	B.D.4.1	2	100%
					B.D.E.1	1	2%
211	S211N	78	48	30	B.D.4.1.1	1	33%
					B.D.E.1	57	100%
					B.D.E.1.1	57	100%
					B.D.E.1.2	4	100%
					B.D.E.1.7	12	100%
					B.D.E.5+	1	100%
276	S276N	21	0	21	A.D	1	50%
					A.D.3.1+	4	100%
					A.D.3.2+	6	100%
					A.D.3.9	10	91%
377	S377N	25	1	24	A.D.1.5	2	25%
					A.D.1.9+	21	100%
					A.D.1.10+	1	100%
					A.D.3.7	1	6%
386	I386M	1	1	0	B.D.E.1	1	2%
389	S389P	75	46	29	B.D.E.1+	56	98%
					B.D.E.1.1	3	100%
					B.D.E.1.2	4	100%
					B.D.E.1.7	12	100%
432	I432V	1	1	0	B.D.E.1	1	2%
445	K445R	1	1	0	B.D.E.1	1	2%
450	V450I	3	0	3	B.D.4.1.1	1	33%
					B.D.E.1	2	4%
466	S466N	1	0	1	A.D.3.7	1	6%
467	L467F	1	1	0	B.D.E.1	1	2%
470	K470R	2	0	2	A.D.1.5	2	25%

Non-synonymous mutations within antigenic regions of the F gene observed in any BMC genome. Each row represents an individual mutation. The first column is the amino acid position within F, and the second column shows the amino acid change observed. The next 3 columns are the number of BMC genomes that had this mutation in the full dataset, the 2023–2024 season dataset, and the 2024–2025 season dataset, respectively. The sixth column lists the lineages in which the mutations were found. The final 2 columns list the number of genomes within that lineage that contained the mutation and what percentage of all BMC genomes of that lineage had the mutation. Mutations that define a lineage are noted with “+”.

### RSV-A Sequences Represent 3 Major Groupings

**Figure 3.**
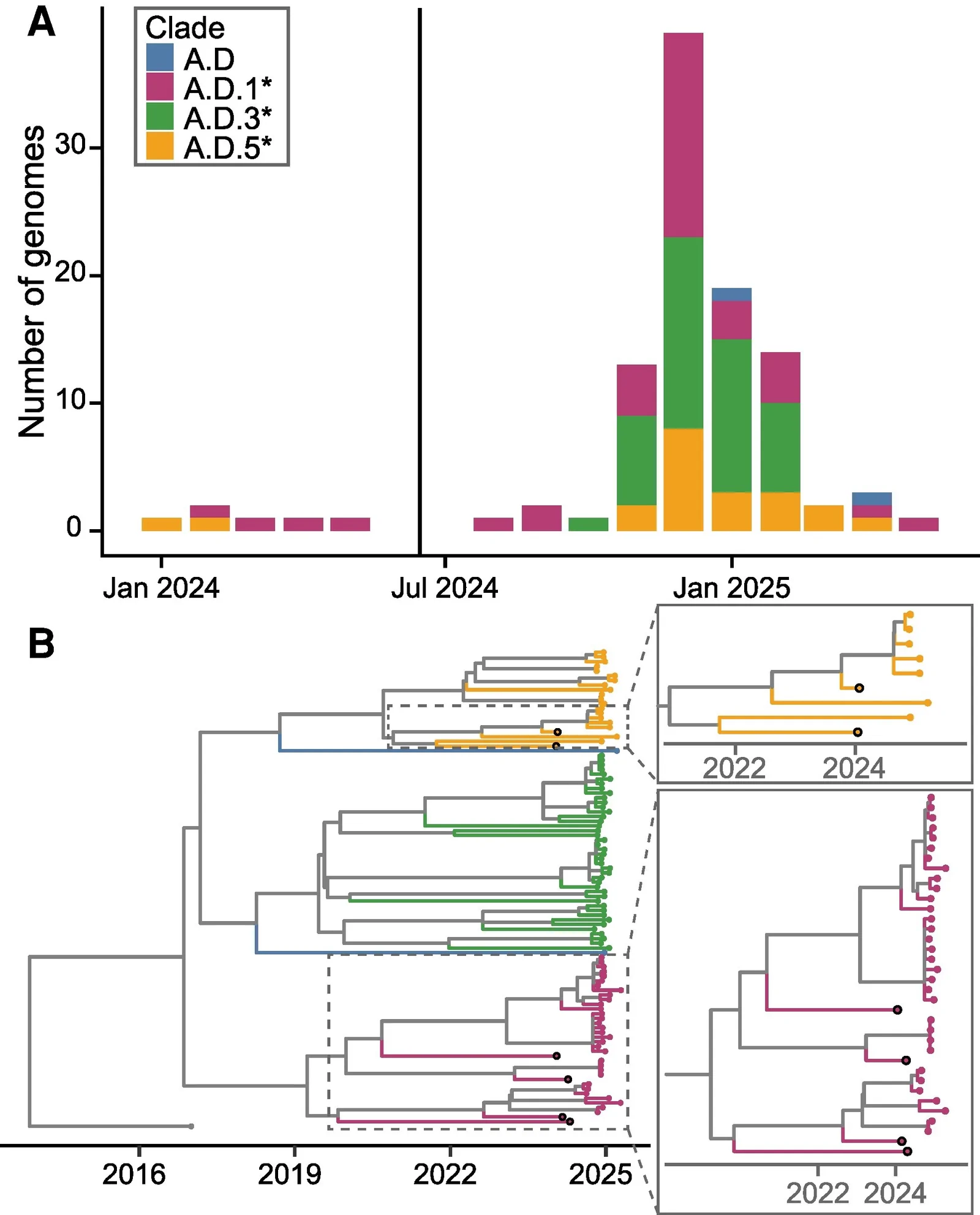
Comparison of major RSV-A clade representation. (*A*) Number of RSV-A genomes sequenced, grouped by sample collection month. Bars are colored by the major grouping of each RSV-A clade; eg, genomes in clades A.D.5.1 and A.D.5.2 are grouped together under the “A.D.5*” major clade (colored in yellow). The vertical line between June and July 2024 represents the transition point between the previously sequenced 2024 samples and the 2024–2025 samples sequenced in this study. (*B*) Time-resolved phylogenetic tree of all RSV-A genomes sequenced from BMC samples. All BMC samples are colored by major clade, as in panel *A*. The reference genome (hRSV/A/England/397/2017) is colored in dark gray. The 6 genomes previously published [27], which were originally collected in January–June 2024, are outlined in black. The dashed boxes over the tree indicate where the inset boxes originate from.

The sequenced RSV-A genomes from BMC were split between 3 major groupings of currently circulating clades: A.D.1* (n = 32), A.D.3* (n = 42), and A.D.5* (n = 19) (Figure 3*A*), with no single clade dominating the RSV-A population sequenced. Within each major grouping, we observed multiple subclades, including A.D.1.5 (n = 7), A.D.1.6 (n = 4), A.D.1.9 (n = 21), A.D.3 (n = 3), A.D.3.1 (n = 4), A.D.3.2 (n = 6), A.D.3.3 (n = 2), A.D.3.7 (n = 16), A.D.3.9 (n = 11), A.D.5.1 (n = 7), and A.D.5.2 (n = 12) (Supplementary Figure 3). Although we had previously observed A.D.1* and A.D.3* genomes in early 2024, the 2024 genomes did not appear to seed ongoing transmission in a time-resolved phylogenetic tree analysis (Figure 3*B*). Within each clade, we identified a genetically diverse set of viruses with recent common ancestors of each of the A.D.1*, A.D.3*, and A.D.5* clades estimated around 2019. These patterns support the hypothesis that RSV-A from many different individual introductions were circulating simultaneously over this period of time, and that the shift in subtype dominance did not result from a single introduction or clade gaining prominence.

### Observation of RSV-B Subclade Expansion

**Figure 4.**
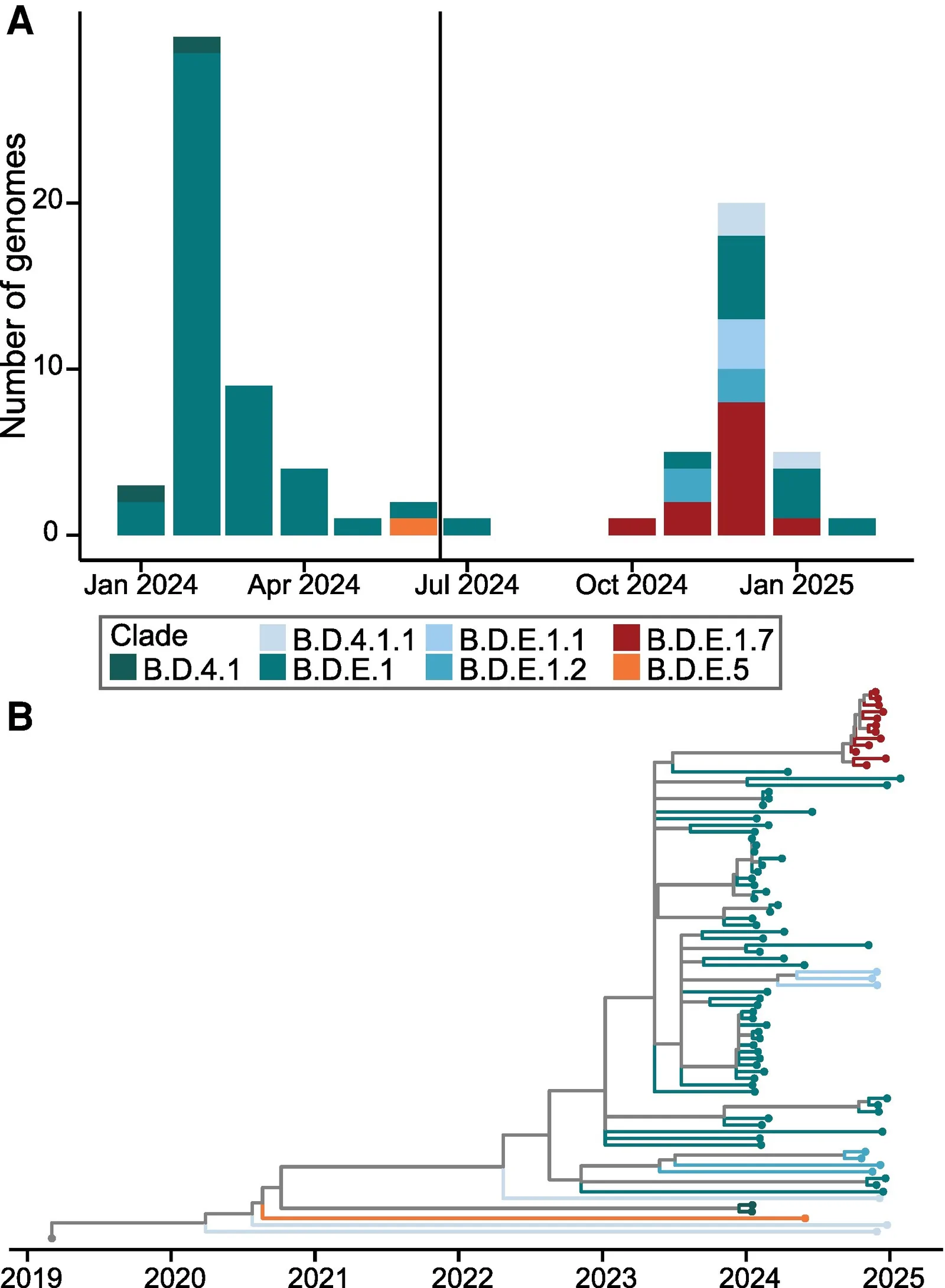
Comparison of RSV-B clade representation. (A) Number of RSV-B genomes sequenced, grouped by sample collection month. Bars are colored by RSV-B clade. The vertical line between June and July 2024 represents the transition point between the previously sequenced 2024 samples and the 2024–2025 samples sequenced in this study. (*B*) Time-resolved phylogenetic tree of all RSV-B genomes sequenced from BMC samples. All BMC samples are colored by major clade, as in panel *A*. The reference genome (hRSV/B/Australia/VIC-RCH056/2019) is colored in dark gray. Genomes originating from samples collected in January–June 2024 were previously published [27].

As in the early 2024 sequences [27], almost all genomes sequenced from BMC belonged to the B.D.E.1 clade (n = 12) or its newly defined sub-lineages: B.D.E.1.1 (n = 3), B.D.E.1.2 (n = 4), and B.D.E.1.7 (n = 12) (Figure 4*A*). As in the RSV-A analysis, a time-resolved phylogenetic tree showed that no single individual genome from early 2024 expanded or was a direct ancestor of 2024–2025 circulating genomes (Figure 4*B*, Supplementary Figure 4). These findings support RSV-B's emergence as a result of multiple independent introductions.

### Local Expansion of Subclade B.D.E.1.7

We were struck by the appearance of a tightly clustered group of 12 genomes classified as B.D.E.1.7, a recently defined sub-lineage from B.D.E.1 defined by G:K32R and G:K223E mutations. B.D.E.1.7 genomes represented approximately one-third of all RSV-B genomes from BMC sequenced in the 2024–2025 season. When comparing clade proportions to other RSV genome sequencing across the United States, B.D.E.1.7 was primarily observed within the northeast (Supplementary Figure 5A); 24 of 34 B.D.E.1.7 genomes identified in the 2024–2025 season originated from Massachusetts.

**Figure 5.**
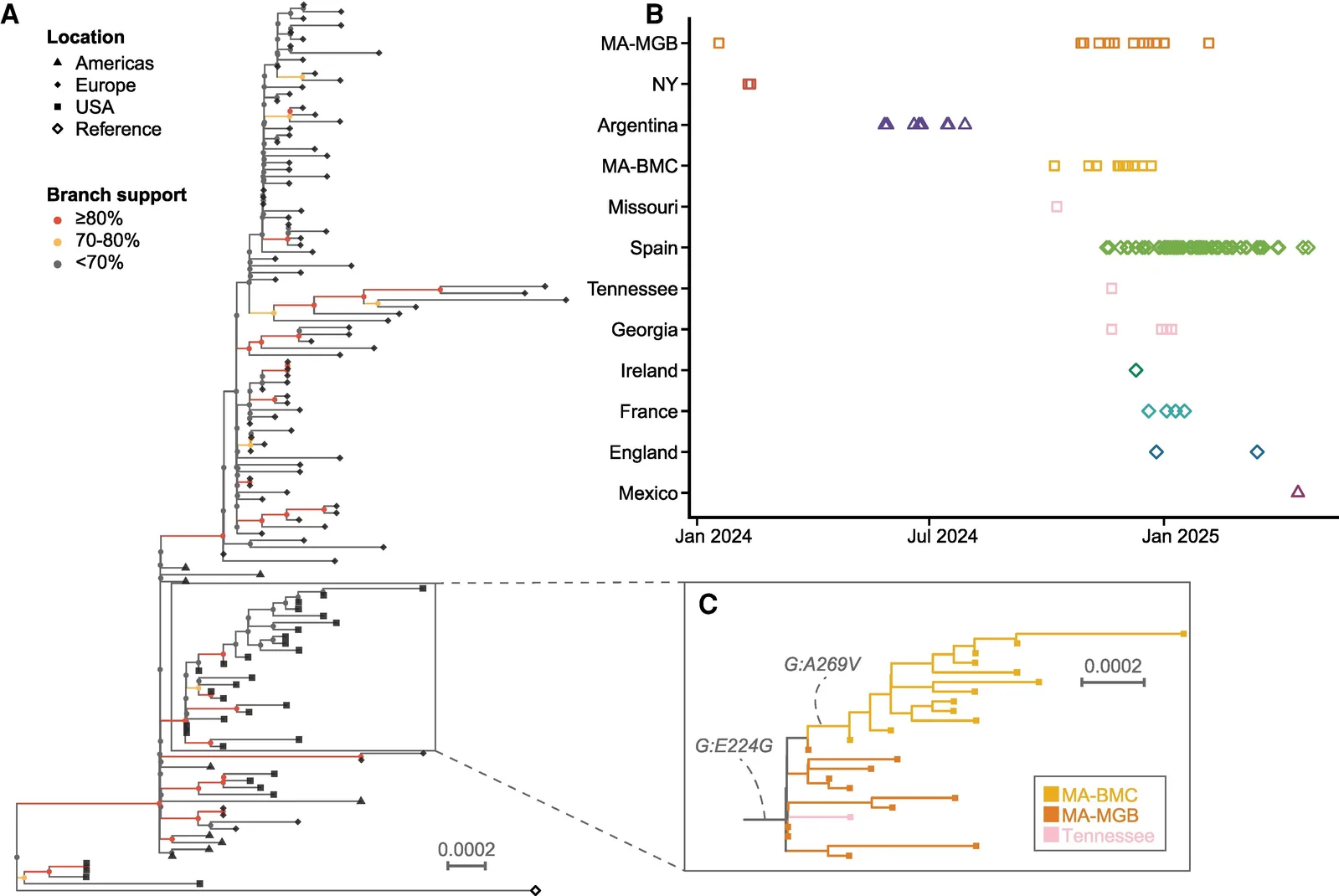
B.D.E.1.7 clade emergence. (*A*) Maximum-likelihood phylogenetic tree of all B.D.E.1.7 genomes. Branch tips are shaped by sample origin location: triangles for samples from the Americas (outside of the United States), circles for samples from Europe, and squares for samples from the United States. A B.D.E.1 reference genome (hRSV/B/USA/2022A7421/2022) has an open diamond tip. Inner nodes are colored by bootstrap support. (*B*) Sample collection dates for publicly available B.D.E.1.7 genomes, colored by sample origin location and shaped by location to match tip shapes in panel *A*. Each point represents a single sample/genome. (*C*) An expansion of part of the larger tree in panel *A*. Genomes are colored by location, matching colors in panel *B*. Amino acid changes that differentiate branches of the tree, derived from Nextclade, are labeled. Dashed lines connect amino acid changes to some branches for visualization. Abbreviations: MA-BMC, Massachusetts from Boston Medical Center (this study); MA-MGB, Massachusetts from Mass General Brigham; NY, New York.

We expanded the exploration of B.D.E.1.7 genomes globally (Figure 5*A*) to better understand where this clade has been observed, finding that the first 3 genomes assigned to B.D.E.1.7 originated in New York and Massachusetts in early 2024, followed by Chile and Argentina, then appeared in both Massachusetts sampling sites throughout late 2024 (Figure 5*B*). Over the winter, B.D.E.1.7 began appearing in Spain, with a scattering of genomes originating elsewhere. We aligned these global B.D.E.1.7 genomes and created a maximum-likelihood phylogenetic tree (Figure 5*A*), which showed further within-clade groupings broadly separating genomes from Europe from those from the Americas despite the highly similar genomes overall. The original 3 genomes (and the one from Missouri) were distinct from all 2024–2025 genomes, which contained 2 additional non-synonymous mutations: G:*311Q, a 7-amino acid extension to the G protein, and L:S1320P, within the capping domain of L (Supplementary Figure 5B).

Noting that the lineage-defining mutations for this clade are within the G gene, we looked for evidence of selection within the G gene on the B.D.E.1.7 branch of the RSV-B maximum-likelihood tree of genomes from BMC (Supplementary Figure 4). FEL analyses with the B.D.E.1.7 branch specified identified 4 amino acids under pervasive positive diversifying selection within G (Supplementary Table 4): K32R, T139I, E224G, and K256N. Three of these 4 amino acids were also identified by MEME analyses with the B.D.E.1.7 branch specified as episodic diversifying positive selection. Of these identified sites, G:K32R is a lineage-defining mutation for the B.D.E.1.7 clade, and both G:T139I and G:E224G are exclusive to B.D.E.1.7 genomes within the BMC genomes sequenced. G:K256N appears in 9 of the 12 B.D.E.1.7 BMC genomes.

Looking at these mutations in the global B.D.E.1.7 dataset, G:K32R and G:T139I are present in all genomes. G:K256N is present in all except for the 3 of 12 BMC genomes noted above. Interestingly, G:E224G is specific to all Massachusetts genomes and the 1 Tennessee genome. Within this group of genomes, we found that only the BMC genomes contained an additional non-synonymous mutation, G:A269V (Figure 5*C*). This mutation is not found in any other B.D.E.1.7 genomes, but it is not exclusive to B.D.E.1.7 genomes within the BMC genomes sequenced. Within the BMC genome set, G:A269V also appears in genomes from clades B.D.4.1.1 (n = 1), B.D.E.1 (n = 19), B.D.E.1.1 (n = 3), and B.D.E.1.2 (n = 4). These observations of the newly emergent B.D.E.1.7 clade suggest highly localized adaptation of particular clades and within-clade diversity.

## DISCUSSION

One key finding from this work is the shifting seasonal dominance between RSV subtypes. Over 4 years of sequencing in the Boston area [26, 27], there have been switches from RSV-A dominance in 2022 to RSV-B dominance in the 2023–2024 season, followed by RSV-A dominance in the 2024–2025 season. The clinical outcomes were similar between RSV-A and RSV-B-positive patients in this study. A total of 75% of the samples containing RSV-A were from samples collected at emergency department visits or inpatient care, compared to 69% for RSV-B.

The shifting seasonal dominance between RSV subtypes is consistent with other longitudinal RSV sequencing studies. Strong shifts in RSV-A and B dominance occurred regularly on a national level in China [51]. A strong annual shift in RSV-A and B dominance was also noted between 2022 and 2023 in a recent epidemiological study that leveraged samples collected in Chicago, IL [52]. It is notable that this pattern of strong RSV-A/B subtype shifts is not always observed and that some locations in the United States (eg, Minnesota) show a more even distribution of RSV-A and B year-over-year [17].

In our study, neither RSV subtype showed evidence of strains from previous seasons persisting into the 2024–2025 season. These results are consistent with modeling studies suggesting that RSV-A and B are annually introduced into a region through air travel and that local factors then determine spread and evolution over the season [53].

A strong example of this outside introduction (likely by air travel) followed by expansion is the transmission of subclade B.D.E.1.7 in the 2024–2025 season. The B.D.E.1.7 expansion in Boston is seen a few months after the appearance and expansion of this same sub-lineage in Argentina. The shared mutations between the 2 geographically distant transmission events provide strong phylogenetic support for the hypothesis that the Boston genomes are derived from the same ancestor as the Argentine B.D.E.1.7 circulation, distinct from the early 2024 genomes from the northeast United States. While the observation of B.D.E.1.7 in Boston predates its appearance in Europe, the phylogenetic tree suggests this expansion was more likely from this common ancestor than from Massachusetts directly. This analysis supports the hypothesis that new sub-lineages with transmission-enabling mutations can rapidly expand through rapid global transport.

We observed multiple mutations in this transnational transmission that are not present in the original sub-lineage definition. In addition to point mutations in the attachment protein (G), we observed a non-synonymous change in the polymerase (L), which alters a serine within the capping domain to a proline. Additionally, we note that almost all B.D.E.1.7 genomes contain a 7-amino acid C-terminal extension of the G protein. This extension has been noted in several earlier genome sequencing studies and was associated with severe disease in one case series [54, 55]. The repeated appearance (and apparent subsidence) of this C-terminal tail in RSV-B suggests it provides a transmission advantage that is worthy of further study.

The significant expansion of B.D.E.1.7 in Boston showed evidence of compartmentalized evolution following the initial introduction. All Massachusetts genomes and 1 Tennessee genome contained the G:E224G mutation, which separated these sequences from other sub-lineage isolates in other locations. Samples from BMC all contained an additional G:A269V mutation that distinguished these genomes from those collected at other Massachusetts sites. This mutation separates genomes collected over the same time period, indicating simultaneous non-overlapping transmission patterns of RSV-B in this urban environment.

Recent studies have documented the appearance of nirsevimab-resistant RSV causing breakthrough infections in antibody-treated children—particularly within RSV-B clinical cases [10]. Deep mutational scanning of nearly all possible mutations to the F protein has identified sporadic appearances of antibody-resistant strains in genomic surveillance, but these mutations are not widespread [50]. We do not find evidence of prophylactic-resistant mutations in our collected genomes, but their presence in other countries argues for continued genomic sequencing to identify if/when resistant viruses begin circulating in this area.

Like all studies involving clinical samples, this study was limited by the sampling strategy (not all possible samples were collected) and by which samples had high enough viral load to allow for sequencing that met the quality thresholds for analysis. It is possible that the subset of samples that we receive for processing is not fully representative of the population of RSV+ samples, though our comparisons of sample dates, patient age, and patient sex suggest that the metrics that we are able to compare are representative of the larger population. Additionally, sampling and surveillance strategies vary widely across the globe, which makes global tracking of specific lineages more challenging. Despite these limitations, our results match well with samples and sequencing done independently in the same area.

This work adds to the growing body of RSV genomic surveillance and allows for direct comparisons to be made within one location across time, in comparison to the 2023–2024 BMC genomes. The expansion of genomic surveillance also allows for geographic comparisons to other genomes from the 2024–2025 season, even from the same city. This sequencing sheds light on the expansion of geographically restricted RSV sub-lineages, an increasingly observed phenomenon among RSV surveillance efforts [18, 28]. Following the transmission of these sub-lineages over time can help understand how this virus moves around the world and spot troubling variants and prophylactic-resistant mutants early. As RSV interventions continue to be developed, surveillance will be increasingly important for understanding any selective pressures toward interventions, as well as any changes in transmission dynamics.

## Supplementary Material

ofag564_Supplementary_Data

## Supplementary Data

Supplementary materials are available at the Journal of the Pediatric Infectious Diseases Society online (http://jpids.oxfordjournals.org). Supplementary materials consist of data provided by the author that are published to benefit the reader. The posted materials are not copyedited. The contents of all supplementary data are the sole responsibility of the authors. Questions or messages regarding errors should be addressed to the author.
