# Supplementary material for: Genomic Surveillance Uncovers a Shift in Respiratory Syncytial Virus Subtype Dominance and the Local Expansion of Subclade B.D.E.1.7
Source: Open Forum Infect Dis. 2026 Sep 9;13(9):ofag564. doi: 10.1093/ofid/ofag564 (PMC13579484; doi:10.1093/ofid/ofag564)
Supplement: Supplementary file 1 [file ofag564_supplementary_data.zip › Revision - Supplemental.pdf]

Supplemental Information: Genomic surveillance uncovers a shift in respiratory syncytial virus subtype dominance and the local expansion of subclade B.D.E.1.7

Author Information

Emily LaVerriere, PhD[1,2]*, Sasha Behar, BA[1,2]*, Yan Mei Liang, BA[1,3], Marisa Nielsen, PhD[3,4], Manish Sagar, MD[1,3], John H. Connor, PhD[1,2]

Affiliations:

1. Department of Virology, Immunology and Microbiology, Boston University Chobanian & Avedisian School of Medicine
2. National Emerging Infectious Diseases Laboratories, Boston University
3. Department of Medicine, Boston University Chobanian & Avedisian School of Medicine
4. Clinical Microbiology, Boston Medical Center; Department of Pathology and Laboratory Medicine, Boston University Chobanian & Avedisian School of Medicine

* These authors contributed equally to this work.

Corresponding author: John H. Connor. 620 Albany St., Boston, MA 02118, jhconnor@bu.edu

Table of Contents

Supplemental Figures....2
Supplemental Figure 1. Comparison of demographics of samples at BMC to national trends... 2
Supplemental Figure 2. RSV+ samples from BMC.... 3
Supplemental Figure 3. Comparison of all clades of RSV-A.... 4
Supplemental Figure 4. Genetic distances between RSV-B genomes sequenced.... 5
Supplemental Figure 5. B.D.E.1.7 detection within the United States.... 6
Supplemental Tables (titles and captions).... 7
Supplemental Table 1. All data accession numbers for genomes generated in this study.... 7
Supplemental Table 2. Background genomes used in analyses.... 7
Supplemental Table 3. Number of RSV-A and RSV-B genomes sequenced across the US from 2024-2025 season.... 7
Supplemental Table 4. Selection analyses on the RSV-B G protein.... 7
References.... 7

Supplemental tables are in a separate spreadsheet, though their titles and captions are included here.

Supplemental Figures

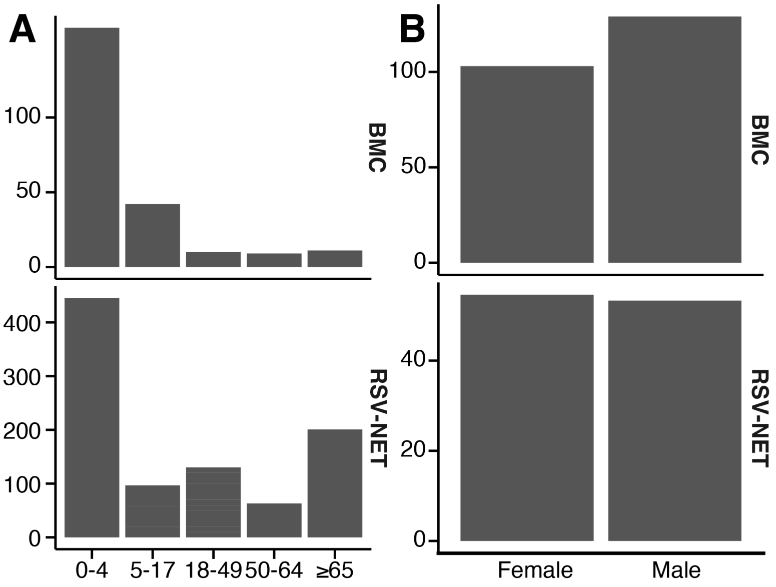

Supplemental Figure 1. Comparison of demographics of samples at BMC to national trends. (A) Comparison of patient age distribution in BMC samples received ("BMC") and demographics from all states in RSV-NET ("RSV-NET"). (B) Comparison of patient sex distribution in BMC samples received ("BMC") and demographics from all states in RSV-NET ("RSV-NET"). In both panels, y-axes denote number of samples in each group (BMC) or the rate of cumulative rate of hospitalizations per 100,000 people over the 2024-2025 season, per age group (RSV-NET).

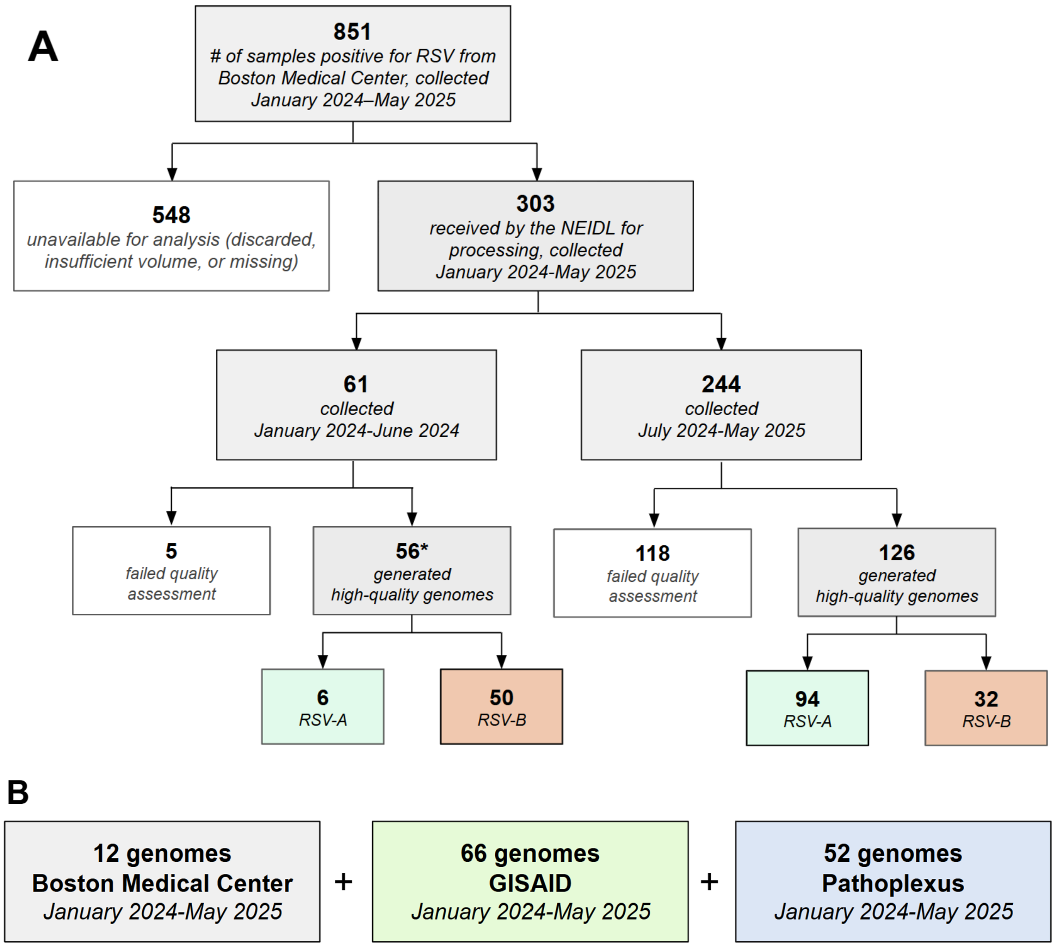

Supplemental Figure 2. RSV+ samples from BMC.
(A) Processing and analysis pipeline showing the number of RSV+ samples that went through each step. *: 54 high-quality genomes were originally reported from January - June 2024 [1]. Two additional samples collected during this time period were processed with the second set of samples (July 2024-May 2025), and resulted in 2 additional RSV-B genomes, bringing the total number of high-quality genomes from January-June 2024 up to 56. (B) Subset of focal genomes and background genomes used for the B.D.E.1.7 clade-specific analysis (**Figure 5**). The focal genomes were the 12 B.D.E.1.7 genomes from the BMC sequencing, and the background set were the 66 genomes from GISAID and 52 genomes from Pathoplexus (all B.D.E.1.7), which are detailed in **Supplemental Table 2**.

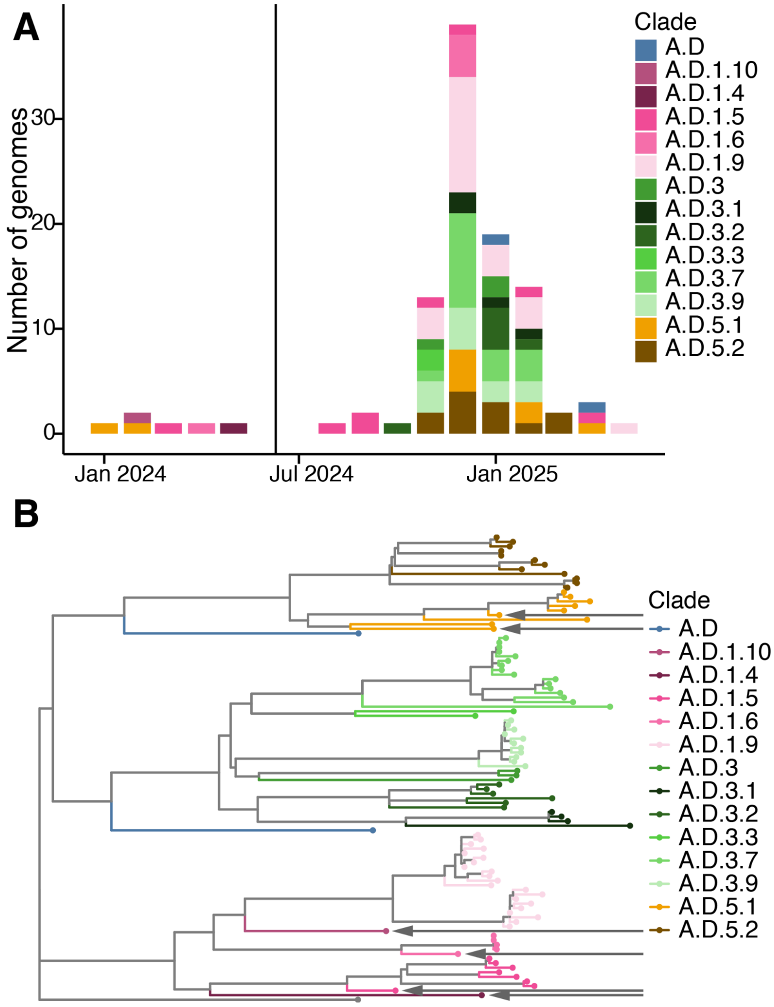

Supplemental Figure 3. Comparison of all clades of RSV-A.
(A) Number of RSV-A genomes sequenced, grouped by sample collection month. Bars are colored by RSV-A clade. The vertical line between June and July 2024 represents the transition point between the previously sequenced 2024 samples [1] and 2024-2025 samples sequenced in this study. (B) Maximum-likelihood phylogenetic tree of all RSV-A genomes sequenced from BMC samples. All BMC samples are colored by clade, as in panel (A). The reference genome (hRSV/A/England/397/2017) is colored in dark gray. Arrows note the six genomes previously published.

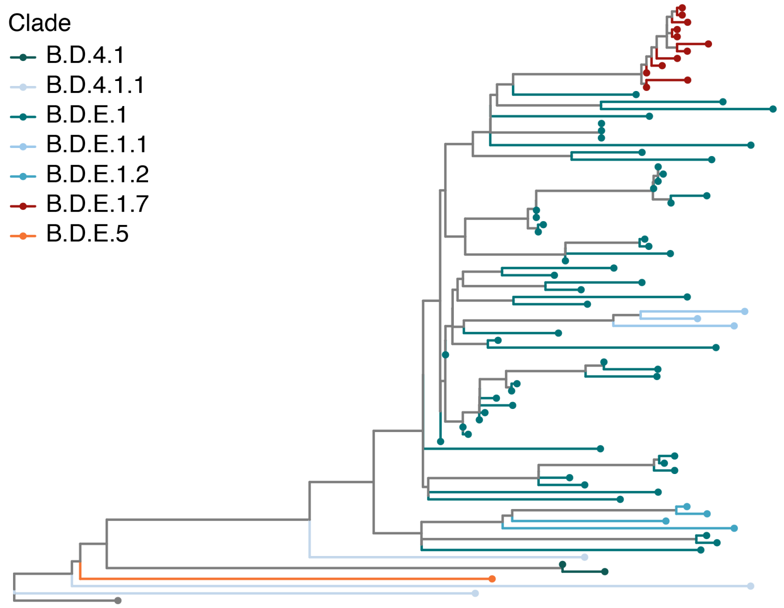

Supplemental Figure 4. Genetic distances between RSV-B genomes sequenced.
Maximum-likelihood phylogenetic tree of all RSV-B genomes sequenced from BMC samples. All BMC samples are colored by RSV-B clade, as in panel (A). The reference genome (hRSV/B/Australia/VIC-RCH056/2019) is colored in dark gray. Genomes originating from samples collected in January-June 2024 were previously published [1].

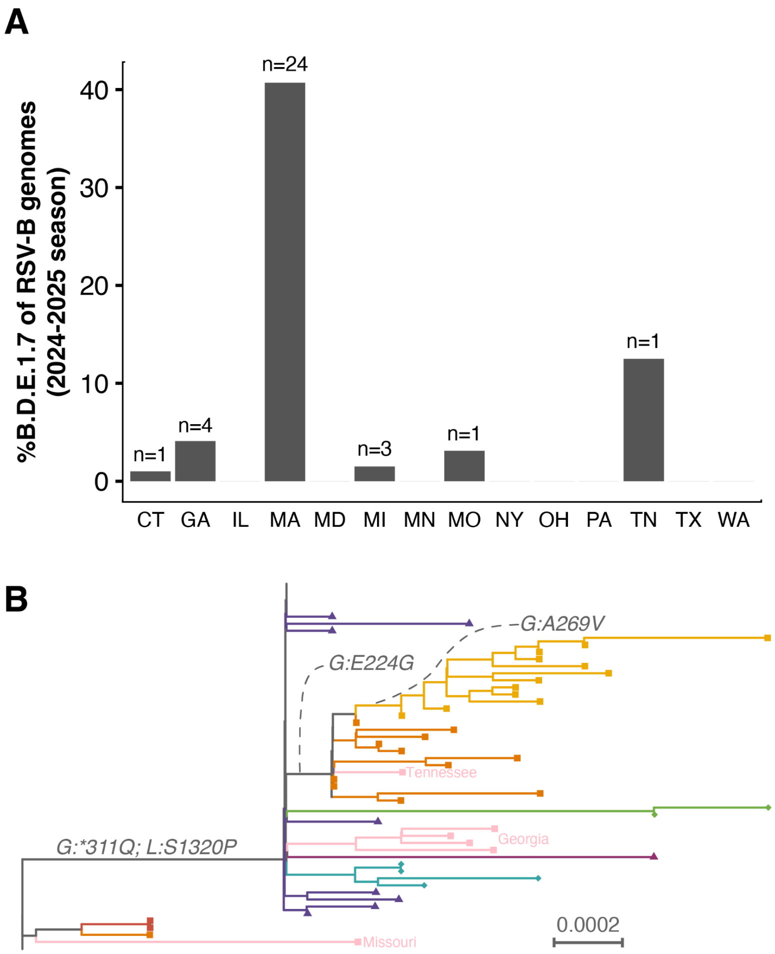

Supplemental Figure 5. B.D.E.1.7 detection within the United States.
(A) Percent of sequenced RSV-B genomes assigned to clade B.D.E.1.7 for United States locations from July 2024-September 2025. States with B.D.E.1.7 detections included: Connecticut (n=1/103), Georgia (n=4/97), Massachusetts (n=24/59), Michigan (n=3/194), Missouri (n=1/32), and Tennessee (n=1/8). All other states had no B.D.E.1.7 genomes detected over this time period. Note - the two B.D.E.1.7 genomes and one additional Massachusetts genome were detected before the 2024-2025 season, and they are not included in these summary data. They are present on both the full tree, and in panel B of this figure. (B) An expansion of part of the larger tree in Figure 5. Genomes are colored by location, matching those in **Figure 5B**. Amino acid changes that differentiate branches of the tree, derived from nextclade, are labeled. Dashed lines connect amino acid changes to some branches for visualization.

Supplemental Tables (titles and captions)

Supplemental Table 1. All data accession numbers for genomes generated in this study.
Genomes labeled as “previously published” are from early 2024 sequencing from BMC [1]. Median depth refers to the median of read-depth across all genomic positions. % genome coverage refers the percentage of genomic positions which had at least 20x depth, out of all genomic positions. Genomes can be found in Pathoplexus SeqSet PP_SS_3037.1 [2].

Supplemental Table 2. Background genomes used in analyses.
List of all genome sequences used in background analysis for **Figure 5**. Genomes can be found in either Pathoplexus SeqSet PP_SS_3070.1 or GISAID EPI_SET_26032ht. Focal genomes in this analysis were the 12 B.D.E.1.7 genomes within the BMC set, which are included in the Pathoplexus SeqSet [3].

Supplemental Table 3. Number of RSV-A and RSV-B genomes sequenced across the US from 2024-2025 season.
Comparison of RSV-A and RSV-B genomes sequenced across the United States originating from samples from the 2024-2025 season. All numbers come from either published genomes reported (Connecticut [4], Georgia [5,6], Illinois [7], Maryland [8], Minnesota [9]) or from genomes publicly available (Michigan [10], Missouri [11], New York [11], Ohio [11], Pennsylvania [11], Texas [11], and Washington [11,12]). Massachusetts genomes include those in this study and publicly available genomes [13].

Supplemental Table 4. Selection analyses on the RSV-B G protein.
Analysis of selection pressure on specific sites within the RSV-B G protein. Both FEL and MEME were run on all RSV-B genomes sequenced from BMC, with the B.D.E.1.7 branch set as the focal branch. Sites that were significantly called in each analysis (default p-value threshold of 0.1) are noted with * next to the p-value. Substitutions that were significant in at least one set of analyses (FEL or MEME) and were present in more than one genome within the B.D.E.1.7 branch are shown.

References

1. LaVerriere E, Behar S, Sher-Jan C, Liang YM, Sagar M, Connor JH. Genomic Epidemiology of Respiratory Syncytial Virus in a New England Hospital System, 2024. Open Forum Infect Dis. **2025**; 12(6):ofaf334.

2. SeqSet: BMC/NEIDL RSV genomes, 2024-2025 season [Internet]. Pathoplexus; 2026. Available from: https://pathoplexus.org/seqsets/PP_SS_3037.1

3. SeqSet: BMC/NEIDL RSV B.D.E.1.7 analysis [Internet]. Pathoplexus; 2026. Available from: https://pathoplexus.org/seqsets/PP_SS_3070.1

4. Kwon J, Vries EM de, Lemey P, et al. Genomic surveillance of a deeply sampled local population reveals age-specific drivers of RSV transmission [Internet]. medRxiv; 2026. Available from: https://www.medrxiv.org/content/10.64898/2026.05.07.26350887v1

5. Smith K, Martinez J, Yu H, et al. Development and Evaluation of an ARTIC-Based Amplicon Sequencing Assay for Whole-Genome Characterization of Respiratory Syncytial Virus [Internet].

medRxiv; 2026. Available from:
https://www.medrxiv.org/content/10.64898/2026.04.06.26350258v1

6. Rachida S, Ahmed A, Rojas-Gallardo D, et al. Human Respiratory Syncytial Virus in Vaccinated and Unvaccinated Adults, Georgia, USA, 2024–2025. Emerg Infect Dis. **2026**; 32(5):827–829.

7. Rios-Guzman E, Almohtadi R, Borrowman SH, et al. Functional Characterization of RSV Clades Associated with Prophylactic Breakthrough Infections in Pediatric Transmission Clusters [Internet]. bioRxiv; 2026. Available from: https://www.biorxiv.org/content/10.64898/2026.05.29.728867v1

8. Zhuang TX, Fall A, Norton JM, et al. Whole-genome sequence characterization of respiratory syncytial virus in the Johns Hopkins Health System during the 2024–2025 respiratory season. Microbiol Spectr. **2025**; 13(11):e02065-25.

9. Evans D, Kunerth H, Mumm E, et al. Expanded Genomic Surveillance and Seasonal Comparisons of Human Respiratory Syncytial Virus Infections in Minnesota, USA, 2023-2025. Influenza Other Respir Viruses. **2026**; 20(5):e70257.

10. Lauring, A. S.; Papalambros, L. K. University Of Michigan, Internal Medicine - Infectious Disease. *Accessed via Pathoplexus.*

11. S. Sabin, A. Kelleher, X. Lu, P. R. Lourenco, P. Mandal, C. Smith, P. W. Cook, C. R. Paden & L. Atherton. Centers for Disease Control and Prevention, Coronaviruses and Other Respiratory Viruses Division. *Accessed via Pathoplexus.*

12. Roychoudhury, P.; Xie, X.; Sereewit, J.; Ellis, S.; Greninger, A. University of Washington, Laboratory Medicine and Pathology. *Accessed via Pathoplexus*.

13. Paull, J.; Messer, K.; Adams, G.; Nabatanzi, D.; Dobbins, S.; Tomkins-Tinch, C.; Mboowa, G.; Luebbert, L.; Worby, C.; Wewior, N.; Samani, P.; DeRuff, K.; Blumenstiel, B.; Moreno, G.; Earl, A.; Gnirke, A.; MacInnis, B.; Sabeti, P.; Lemieux, J.; Park, D. Broad Institute of MIT and Harvard, Infectious Disease and Microbiome Program. *Accessed via Pathoplexus.*
